# Significance Test and Genome Selection in Bayesian Shrinkage Analysis

**DOI:** 10.1155/2010/893206

**Published:** 2010-06-10

**Authors:** Xiaohong Che, Shizhong Xu

**Affiliations:** ^1^Department of Statistics, University of California, Riverside, California 92521, USA; ^2^Department of Botany and Plant Sciences, University of California, Riverside, California 92521, USA

## Abstract

Bayesian shrinkage analysis is the state-of-the-art method for whole genome analysis of quantitative traits. It can estimate the genetic effects for the entire genome using a dense marker map. The technique is now called genome selection. A nice property of the shrinkage analysis is that it can estimate effects of QTL as small as explaining 2% of the phenotypic variance in a typical sample size of 300–500 individuals. In most cases, QTL can be detected with simple visual inspection of the entire genome for the effect because the false positive rate is low. As a Bayesian method, no significance test is needed. However, it is still desirable to put some confidences on the estimated QTL effects. We proposed to use the permutation test to draw empirical thresholds to declare significance of QTL under a predetermined genome wide type I error. With the permutation test, Bayesian shrinkage analysis can be routinely used for QTL detection.

## 1. Introduction

Interval mapping [1] and multiple interval mapping [2] are the most commonly used methods for QTL mapping. These methods are developed in the maximum likelihood framework, which has limitation in terms of handling large saturated models. Bayesian mapping [3–7] deals with large models more efficiently through the reversible jump Markov chain Monte Carlo (RJMCMC) [4], the shrinkage analysis [8, 9], or the stochastic search variable selection (SSVS) [10]. Shrinkage mapping and SSVS are more efficient in terms of whole genome evaluation because they are statistically easy to understand and also provide better chance to evaluate the entire genome. These two methods are related to the Lasso method for regression analysis [11]. Rather than deleting nonsignificant QTL explicitly from the model, these methods use a special algorithm to shrink estimated QTL effects to zero or close to zero. A QTL with zero estimated effect is treated the same as being excluded from the model. No statistical test is required because genome regions bearing no QTL often show no bumps (QTL effects) in the QTL effect profile (plot of QTL effects against genome location). The visual inspection on the QTL effect profile is not optimal because small QTL may come and go during the MCMC sampling process. It is desirable to provide some kind of statistical confidence on these small QTL. 

Permutation test [12] itself is not a method of QTL mapping; rather, it is a method to find the critical value used to declare the significance of QTL for any method of QTL mapping. It is very efficient in interval mapping under the maximum likelihood framework. A new resampling method was developed by Zou et al. [13] for significance test under the composite interval mapping or other multiple effect based QTL mapping schemes. The new resampling method is computationally less intensive and may perform better than the permutation test. However, it has not been as popular as we would have thought. The reason for this is perhaps due to the fact that the theory behind the method is not straightforward to most QTL mapping experimentalists. The permutation test, although time consuming, does not require any theory and is easy to understand. People tend to trust a simple method they understand, rather than a comprehensive method they do not, even if the simple method is suboptimal. Therefore, the permutation test remains the most popular method for finding the critical value of a test statistic for QTL detection. Kopp et al. [14] applied the permutation test to determine empirical thresholds for Bayesian shrinkage mapping. The problem with such a test for the MCMC implemented Bayesian mapping is the heavy computational burden. Each MCMC run may take one or a few hours to complete for a reasonable sample size of the mapping population. Performing thousands of permutation analyses is not realistic for the Bayesian method. Therefore, improvement of the permutation test applied to Bayesian analysis is required. This is the first objective of this study. 

Broman and Speed [15] treated multiple QTL mapping as a model (variable) selection problem and developed a new method called BIC_*δ*_. More recently, Manichaikul et al. [16] extended the Broman and Speed [15] model selection by allowing epistatic (nonallelic interaction) effects to be included in the model. They called the extended model selection method the penalized LOD score method (pLOD). Two versions of the penalized LOD score method were investigated; one is called the heavy penalized LOD score (pLOD_H_) and the other is called the light penalized LOD score (pLOD_L_). With this new notation, the original BIC_*δ*_ of Broman and Speed [15] was renamed as pLOD_a_, penalized LOD score for additive effects only. The authors compared these methods along with two other BIC-based methods and the Bayesian model selection method of Yi et al. [17] using both simulated data and real data. They concluded that the pLOD methods including epistatic effects and the Bayesian model selection method outperformed other methods in most cases they evaluated.

The model selection methods are alternative method of QTL analysis. They cannot replace the Bayesian shrinkage analysis because the two have quite different purposes. Model selection aims to detecting QTL while Bayesian shrinkage focuses on genome evaluation. We realized that if the Bayesian shrinkage analysis is accompanied with a significance test, it can serve both QTL detection and genome selection. The original Bayesian shrinkage analysis [8, 9] has no significance test associated with the method because the entire genome was evaluated simultaneously in a single model. More recently, researchers, especially animal and plant breeders, became interested in genome selection [18, 19] using the Bayesian method. Applications of genome selection to laboratory mice [20] and human [21] were also reported. Genome selection does not require statistical tests because QTL of the entire genome, regardless the sizes, are included to predict the genomic effect of individuals. However, there is no report so far to investigate whether inclusion of small QTL will benefit genome selection. Cross-validation can be used to determine how large a QTL should be included in genome selection. This is the second aim of this study.

## 2. Methods

### 2.1. Model

For the paper to be self contained, we briefly introduce the Bayesian shrinkage model here. Let *y*_*j*_ be the phenotypic value of a quantitative trait measured from individual *j* for *j* = 1,…, *n*, where *n* is the sample size. Suppose that the individual is genotyped for *m* markers, which are more or less evenly distributed across the genome. Let *X*_*jk*_ be the genotype indicator variable for individual *j* at marker *k* for *k* = 1,…, *m*. The linear model describing the relationship between the phenotype and the genotypes of markers is

(1) $$y_{j}=b_{0}+\overset{m}{\underset{k=1}{\sum }}X_{jk}b_{k}+e_{j},$$

where *b*_0_ is the intercept, *b*_*k*_ is the QTL effect for marker *k*, and *e*_*j*_ is the residual error with an assumed *N*(0, *σ* ^2^) distribution. The reason that the Bayesian shrinkage method can handle a large *m* is the prior distribution assigned to each QTL effect:

(2) $$p\left(b_{k}\right)=N\left(b_{k}\;|\;0,\sigma_{k}^{2}\right),$$

where *σ*_*k*_^2^ is a QTL specific prior variance. This prior alone is not sufficient to generate the desired shrinkage estimate of QTL effect. A hierarchical model with a higher level of prior assignment is necessary, in which the prior variance *σ*_*k*_^2^ is further assigned a scaled inverse chi-square distribution:

(3) $$p\left(\sigma_{k}^{2}\right)=\text{Inv}-\chi^{2}\left(\sigma_{k}^{2}\;|\;\tau ,\omega \right).$$

In the original shrinkage analysis, Xu [9] set *τ* = *ω* = 0, leading to *p*(*σ*_*k*_^2^) = 1/*σ*_*k*_^2^. Ter Braak et al. [22] claimed that this prior is improper and leads to an improper posterior distribution. They revised the prior so that the posterior distribution becomes proper. Their revised prior is

(4) $$p\left(\sigma_{k}^{2}\right)=\text{Inv}-\chi^{2}\left(\sigma_{k}^{2}\;|\;-2\delta ,0\right)\propto \frac{1}{{\left(\sigma_{k}^{2}\right)}^{1-\delta }},$$

where 0 < *δ* ≤ 0.5. If *δ* = 0, this revised prior would be equivalent to Xu's [9] vague prior. However, Xu's vague prior is just excluded from the revised prior. In this study, we used the proper prior of Ter Braak et al. [22], as a precaution to avoid any potential problems caused by the improper posterior distribution of *σ*_*k*_^2^.

### 2.2. Permutation between Markov Chains

In the MCMC-implemented Bayesian shrinkage analysis, Xu [9] plotted the estimated QTL effects against the genome location. We could have plotted a test statistic, say a *t*-test or an *F*-test, against the genome location. Unfortunately, the test statistic requires the posterior standard deviation of each sampled QTL. The empirical posterior standard deviation highly depends on the thinning rate of the Markov chain and thus is always underestimated due to possible autocorrelation. Therefore, we prefer to use the QTL effect profile rather than a test statistic profile. To determine the threshold values for the QTL effects under the null model, we employed a permutation test just like frequentists do in interval mapping [12]. Let *y* = {*y*_*j*_} be the vector of the phenotypic values ordered according to the individuals' natural identification numbers, that is, the original dataset where the individuals' phenotypes match their marker genotypes in the files. Let y* = {*y*_j_*} be a randomly rearranged vector of phenotypes, called a permutation, in which the phenotypes do not match the marker genotypes. Performing a Bayesian shrinkage analysis on the permuted data by running a Markov chain with a desired length, we obtain a posterior sample for all the parameters. For the parameters of interest, say the QTL effects, we record their values and save them in a file as one observation from one permutation analysis. The permutation analysis is repeated independently for a thousand times; we then obtain a thousand observations for each of the interested parameters (QTL effects). This sample contains observations from the empirical distribution of the null model (no QTL effects). The 0.5 *α* × 100% and (1 − 0.5 *α*) × 100% percentiles of a parameter in the thousand permuted samples are the empirical critical values used to declare statistical significance for a QTL in the analysis of the original dataset (phenotypes match the genotypes). This permutation strategy was first applied by Kopp et al. [14]. This so-called “permutation outside the Markov chain” approach is the traditional application of the permutation test [12] to the Bayesian analysis. The problem with this strategy is the extensive CPU time. Each MCMC run may take an hour or so and a complete permutation experiment consisting of 1000 permutation analyses may take a month computing time. Therefore, we will invent a more efficient permutation method to replace this traditional method of permutation.

### 2.3. Permutation within Markov Chain

As the name of the method implies, this permutation strategy permutes the phenotypes in every *h*th iteration within a Markov chain, where 1 ≤ *h* ≤ *L* and *L* is the length of the Markov chain. If *h* = *L*, this approach is equivalent to the permutation-between-chains approach. If *h* = 1, we permute the phenotype in every iteration. The approach is implemented as follows. For each iteration, after all parameters are sampled, the phenotypes are reshuffled before the next round of sampling starts. The total length of the chain is not necessarily longer than a regular Markov chain for the unshuffled data. Therefore, a complete data analysis requires only two chains, one for the original data and one for the reshuffled data. The reshuffled chain provides the 0.5 *α* × 100% and (1 − 0.5 *α*) × 100% percentiles used as critical values of the QTL effects. 

The within-chain permutation is a strategy to generate the posterior distributions of the regression coefficients under the null model. If the genotypes do not match the phenotypes, the Bayesian estimates (posterior means) of the regression coefficients are expected to be zero across all loci. The posterior variances are determined by the residual variance and the variance of the genotypic indicator variables, which are preserved in the permuted sample, regardless how frequent the phenotypes are reshuffled. There is no theory behind this permutation test. We chose this test for the very reason of simplicity. As long as we can control the type I error for the entire genome and produce reasonable powers for all the large QTL, the permutation test should be admissible.

### 2.4. Genome Selection

Genome selection aims to evaluating the genetic effect for the entire genome using dense markers for each individual. When all individuals in a population are evaluated, the genomic effects of different individuals can be compared and the “best” individuals are selected for breeding. How to combine the QTL mapping result with genome selection is an important but not yet answered question. We adopted a five-fold cross-validation test [11] to answer this question. In the cross-validation analysis, we partition the sample into five equal parts (subsamples). Each time, we use four parts (4*n*/5 individuals) to estimate the QTL effects and perform within-chain random shuffling to determine the empirical percentiles for QTL detection. Only significant QTL at the *γ* level is used to predict the total genomic effect for an individual in the remaining part (*n*/5 individuals). Note that the training sample (4*n*/5 individuals) is used for parameter estimation and significance test and the testing sample (*n*/5 individuals) is used for prediction. The squared prediction error (PE) for the s-part is defined as

(5) $$\Delta_{s}\left(\gamma \right)=\frac{5}{n}\overset{n/5}{\underset{j\prime =1}{\sum }}{\left(y_{j^{\prime }}-{\hat{b}}_{0}-\overset{m}{\underset{k=1}{\sum }}X_{kj^{\prime }}{\hat{b}}_{k}\right)}^{2},$$

where *y*_*j*′_ is the phenotypic value of an individual in the test sample and *j*′ indexes all individuals in the test sample. The intercept and the regression coefficients are estimated from the training sample. Note that ${\hat{b}}_{k}^{}$ equals the shrinkage estimate if it passes the thresholds and ${\hat{b}}_{k}^{}=0$ otherwise. The overall PE for the cross-validation test is

(6) $$\text{PE}\left(\gamma \right)=\frac{1}{5}\overset{5}{\underset{s=1}{\sum }}\Delta_{s}\left(\gamma \right).$$

We vary *γ* from 0 to 1 incremented by 0.1. The *γ* value that minimizes the PE is the optimal one used as the criterion of QTL inclusion for genome selection.

## 3. Results and Discussion

### 3.1. Simulation Study

The design of the simulation experiment conducted by Wang et al. [8] was adopted here exepct that the population simulated was an *F*_2_ rather than a BC population. The sample size was fixed at 500, which is a typical sample size used in most QTL mapping experiments. The genome size was 2400 cM long covered by 241 evenly distributed markers (10 cM per marker interval). A total of 20 QTLs were placed on the genome and the positions and effects of the 20 QTL are presented in Table 1. The QTL size varied from 0.3% phenotypic variation to 13% phenotypic variation. The proportions of QTL explaining the total phenotypic variance were calculated based on the following method. The genotype indicator variable for individual *j* at locus *k* is defined as *X*_*jk*_ = {1, 0, −1} for the three genotypes (*A*_1_ *A*_1_, *A*_1_ *A*_2_, *A*_2_ *A*_2_), respectively. Dominance effects were not simulated and also not included in the model for this simulation experiment because they do not help answer questions addressed in this study. These parameter values were used to generate a quantitative trait with a population mean *b*_0_ = 10.0 and a residual error variance *σ* ^2^ = 10.0. The total genetic variance for the trait is

(7) $$V_{G}=\overset{20}{\underset{k=1}{\sum }}\;\overset{20}{\underset{k\prime =1}{\sum }}b_{k}^{}b_{k\prime }^{}\mathrm{cov}\;\left(z_{k},z_{k\prime }\right)=\frac{1}{2}\overset{20}{\underset{k=1}{\sum }}\;\overset{20}{\underset{k\prime =1}{\sum }}b_{k}^{}b_{k\prime }^{}\left(1-2r_{kk\prime }\right),$$

where *r*_*kk*′_ is the recombination frequency between QTL *k* and *k*′, cov (*z*_*k*_, *z*_*k*′_) = var(*z*)(1 − 2*r*_*kk*′_) is the covariance between *Z*_*k*_ and *Z*_*k*′_, and var(*z*) = 1/2 is the variance of *Z* (assuming no segregation distortion). The total genetic variance for the quantitative trait is *V*_*G*_ = *V*_*Q*_ + *V*_*L*_ = 66.384, which is the sum of the genetic variances due to QTL (*V*_*Q*_) and covariance between linked QTL (*V*_*L*_), where

(8) $$\begin{array}{c} V_{Q}=\frac{1}{2}\overset{20}{\underset{k=1}{\sum }}b_{k}^{2}=46.7804,\\ V_{L}=\overset{20}{\underset{k\prime >k}{\sum }}b_{k}^{}b_{k\prime }^{}\left(1-2r_{kk\prime }\right)=19.6034. \end{array}$$

**Figure 1:**
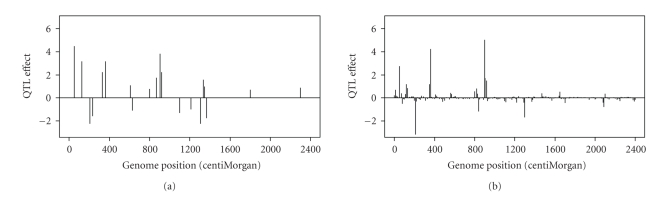
The true and estimated QTL effects for the entire genome of the simulated data. (a) The true positions and effects of the simulated QTL. (b) The estimated positions and effects of QTL using the Bayesian shrinkage method.

The residual error variance for the trait is *σ* ^2^ = *V*_*E*_ = 10.0. Therefore, the total phenotypic variance is *V*_*P*_ = *V*_*G*_ + *V*_*E*_ = 76.384. The proportion of the genetic variance contributed by each QTL is 0.5*b*_*k*_^2^/*V*_*G*_ for the *k*th QTL (given in the column headed with Prop-G in Table 1). The corresponding proportion of the phenotypic variance contributed by the *k*th QTL is 0.5*b*_*k*_^2^/*V*_*P*_ and given in the column headed with Prop-P in Table 1. The true QTL effects are depicted in Figure 1. 

All 241 markers were included in the model, leading to the dimensionality of the model of *n* × (*m* + 1) = 500 × (241 + 1). The burn in period was 1000. The chain was thinned by keeping one observation out of 10 iterations until the posterior sample size reached 5000. The total number of iterations was 1000 + 5000 × 10 = 51000. The true values of the QTL effects and the locations of the simulated QTL are depicted in Table 1.

The true values and estimated values of QTL are depicted in Figure 1. Clearly, the Bayesian shrinkage method provides very reasonable estimates to the true effects. Regions without QTL show no sign of major QTL. For the small QTL, say QTL numbers 19 and 20, the estimated effects are also small with values no larger than the bumps in the no QTL regions (noises). 

**Figure 2:**
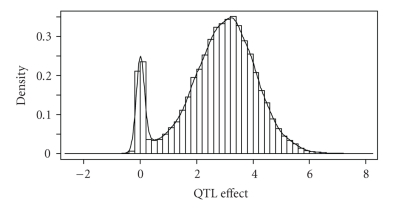
Posterior distribution of QTL number 1 of the simulation experiment. The true effect of the simulated QTL is 4.47. There is a high probability mass at value zero, even though this is the largest QTL out of the 20 QTL simulated.

We calculated the equal tail credible interval at *α* = 0.05, that is, the 2.5%–97.5% percentile range, for each marker. Only one (the largest) QTL was detected because the interval excluded 0 (data not shown). The equal-tail credible intervals of all other QTL covered zero, and thus, they are “not significant” in terms of statistical testing. Using the equal tail credible interval at *α* = 0.10, two more QTLs were detected in addition to the largest QTL (data not shown). Certainly, the equal tail credible interval is not a good criterion for significance test. The posterior distributions for most estimated QTL effects have a special distribution with a spike at zero, which is the cause for the failure of equal tail credible interval as the criterion for significance test. These intervals cannot be used for significance test under the Bayesian shrinkage mapping. The reason is that almost all QTLs have an equal-tail interval covering the null value, for example, zero. Even the largest QTL in our simulation had a high probability mass at zero (see Figure 2). This spike-shaped or zero-inflated posterior distribution for QTL effect is typical in Bayesian shrinkage mapping. If we had used the equal tail interval at *α* = 0.05 as the significance test criterion, only one QTL (the largest one), out of the 20 simulated QTL, would have reached the statistical significance level. The permutation test, however, detected many major QTL, as demonstrated next in the permutation test sections. 

### 3.2. Permutation Outside Markov Chain

**Figure 3:**
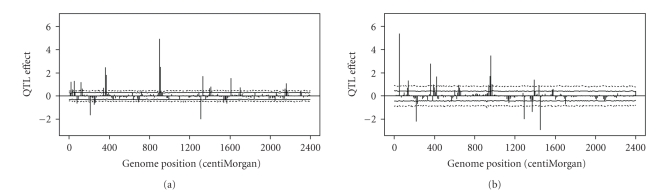
Empirical threshold values generated from permutation analysis and the estimated QTL effects (simulated data)and empirical threshold values generated from permutation analysis at *α* = 0.05 (2.5%–97.5%) and *α* = 0.10 (5%–95%) along with the estimated QTL effects (simulated data). Percentiles for the 2.5%–97.5% interval are plotted against the genome location as dashed lines (wider interval). Percentiles of the 5%–95% interval are plotted against the genome location as solid lines (narrower interval). (a) shows the result of “permutation outside the Markov chain” (b) Result of “permutation within the Markov chain” with phenotype reshuffling in every iteration (*h* = 1).

We generated a total of 5000 permuted samples. Each permuted sample was subject to the same MCMC analysis as the original data (51000 iterations). The SAS/IML program took approximately 20 days in a Dell PC (2.5 GHz and 3.25 Go of RAM). For each marker, the 2.5%–97.5% and 5%–95% intervals (corresponding to *α* = 0.05 and *α* = 0.10) were calculated. The profiles of these percentiles along with the estimated QTL effects are given in Figure 3(a). Using the 2.5%–97.5% interval, we can detect 15 QTL out of the 20 simulated QTL. A few more QTLs with small effects were detected when 5%–95% interval was used. The results here are more reasonable than those when the equal tail credible interval was used. The conclusion is that permutation test applies well to the Bayesian shrinkage mapping. 

### 3.3. Permutation Inside Markov Chain

This permutation strategy only requires running one more chain in addition to the MCMC run of the original data. The phenotypes are reshuffled in every *h*th iteration within the Markov chain. We first evaluated the performance of *h* = 1, that is, reshuffling the phenotype in every iteration. The 2.5%, 5%, 95%, and 97.5% percentiles plotted against the genome location are shown in Figure 3(b) to compare with the result of permutation outside the chains. These intervals (the within-chain permutation) appear to be wider than the intervals of the between-chain permutation analysis. Therefore, the tests for the within-chain permutation are more conservative than the between-chain permutation. Using the within-chain permutation, 13 QTLs were detected for *α* = 0.05 and 19 QTLs were detected for *α* = 0.10, not too much different from the result of the between-chain permutation. A more conservative test is better than a more liberal test, as long as the statistical power is not compromised (examined later in the power study section). 

**Figure 4:**
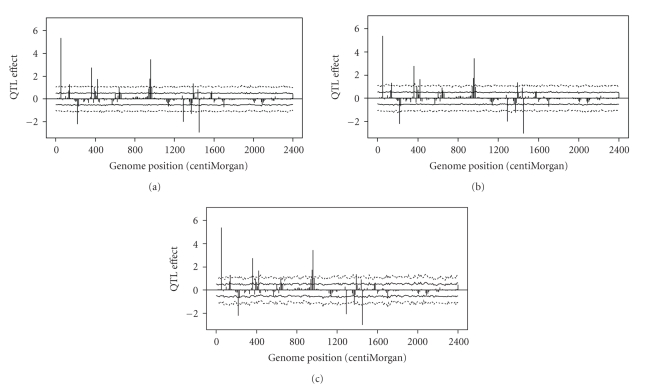
Empirical threshold values generated from “permutation within Markov chain” and the estimated QTL effects (simulated data) and empirical threshold values generated from “permutation within Markov chain” analysis at *α* = 0.05 (2.5%–97.5%) and *α* = 0.10 (5%–95%) along with the estimated QTL effects (simulated data). Percentiles for the 2.5%–97.5% interval are plotted against the genome location as dashed lines (wider interval). Percentiles of the 5%–95% interval are plotted against the genome location as solid lines (narrower interval). (a) Phenotype reshuffling in every 5 iterations (*h* = 5). (b) Phenotype reshuffling in every 10 iterations (*h* = 10). (c) Phenotype reshuffling in every 100 iterations (*h* = 100).

We now evaluate situations where *h* is greater than one. This time we chose three different levels, *h* = 5,10, and 100. The 2.5%, 5%, 95%, and 97.5% percentiles plotted against the genome location are shown in Figure 4. These intervals appear to be similar to *h* = 1 except that the higher *h'*s tend to generate rougher percentile profiles. Therefore, *h* = 1 is more preferable than other values of *h*. Hereafter, we chose *h* = 1 for all subsequent analysis.

### 3.4. Power Analysis

**Figure 5:**
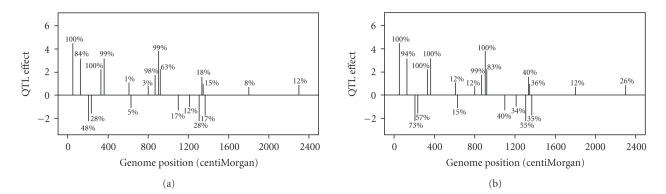
Empirical statistical power for the simulated QTL and empirical statistical powers for the simulated QTL obtained from 100 replicated experiments. (a) Statistical powers at Type I error of *α* = 0.05. (b) Statistical power at Type I error of *α* = 0.10.

Using the same parameters given in Table 1, we simulated 100 more independent samples to investigate the statistical power of the Bayesian shrinkage method. Two MCMC runs were conducted for each sample. One run was the MCMC sampler on the original data to estimate QTL effects and the other run was the MCMC sampler on the within-chain reshuffled data to generate the critical values for QTL detection. The statistical power for each QTL was calculated based on the proportions of samples in which the QTL fell outside the empirical intervals. We observed that if a true QTL failed to be detected at the locus where it was placed, the effect was often picked up by a marker nearby (10 cM away). Therefore, a true QTL was claimed to be detected if one or more of the triplets (three loci) covering the true QTL (20 cM range) was detected. The statistical powers for the 20 QTL are depicted in Figure 5. The powers seem to be reasonable; seven out of the 20 simulated QTL have a power reached 80% at *α* = 0.10. Therefore, the conservative within-chain permutation significance test does not sacrifice much statistical power. 

### 3.5. False Positive Rate

**Figure 6:**
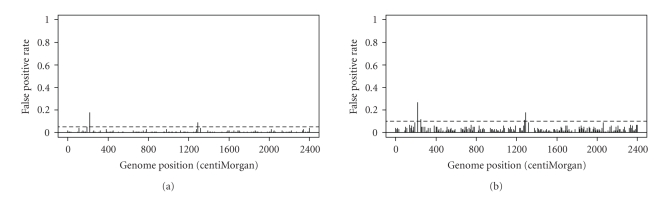
False positive rate profiles for the simulated markers obtained from 100 replicated experiments. (a) False positive rate at *α* = 0.05. (b) False positive rate at *α* = 0.10.

For the 241 marker effects included in the model, 20 × 3 = 60 loci were reserved for the true QTL (20 true QTL plus 40 flanking markers), leaving 241 − 60 = 181 model effects as false QTL. If a false QTL was detected in a particular sample, it was counted as one false positive. For each false QTL, we counted the total number of false positives among the 100 replicated experiments. The proportion of false positive (false positive rate or type I error) was recorded for each false QTL simulated. The false positive rate (FPR) profiles are depicted in Figure 6. Figure 6(a) shows the observed false positive rate when *α* = 0.05. Only two markers had false positive rate larger than the controlled value of 0.05. All other markers had false positive rate less than 0.05. The average false positive rate of all markers was about 0.02. The observed false positive rate is indeed less than 0.05, confirming our previous conclusion that the within-chain permutation approach is conservative. Figure 6(b) shows the observed false positive rate at *α* = 0.10. Only four markers had false positive rates larger than 0.10. The average false positive rate for all these markers was about 0.05, again confirming the conservativeness of the within chain permutation approach. 

### 3.6. Cross-validation for Genome Selection

**Figure 7:**
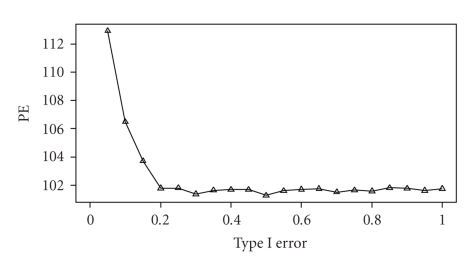
Prediction error (PE) plotted against the Type I error for the simulated data. The squared prediction error (PE) plotted against the Type I error obtained from the fivefold cross-validation test for the simulated data.

Using the original data simulated in the beginning of the experiment (not a sample from the power study), we performed the fivefold cross-validation study to determine how large a QTL should be included in the model to predict the total genetic value of an individual. The PE (squared prediction error) values are plotted against the *γ* value in Figure 7. The minimum PE value occurs at *γ* = 0.2. The decrease of the PE from *γ* = 0.0 to *γ* = 0.2 is very sharp, but after *γ* = 0.2, the PE value tends to be stabilized or slightly increased. The conclusion is that in genome selection, we should choose the *γ* value around 0.2. Of course, this optimal value may vary from sample to sample. We recommend such a cross-validation test for each data analysis to determine how many QTL should be included. From the PE profile, including all QTLs (*γ* = 1) into the prediction model (regardless the sizes of the QTL) does not lead to any significant loss in the precision of genome selection compared to the optimal number of QTL determined by the cross-validation test. Therefore, a robust choice is to include all QTLs in the model for genome selection.

### 3.7. Real Data Analysis

We now use three sample data to demonstrate the application of the permutation test-associated Bayesian shrinkage analysis. These data were collected from QTL mapping experiments in model plants and agricultural crops. 

#### 3.7.1. Arabidopsis Data

The first dataset is the recombinant inbred line data of Arabidopsis data [23], where the two parents initiating the line cross were Bay-0 and Shahdara with Bay-0 as the female parent. The recombinant inbred lines were actually *F*_7_ progeny of single seed descendants of the *F*_2_ plants. The residual heterozygosity was low [23]. Flowering time was recorded for each line in two environments: long day (16-hour photoperiod) and short day (8-hour photoperiod). We used the short day flowering time as the quantitative trait for QTL mapping. The two parents had very little difference in short day flowering time. The sample size (number of recombinant inbred lines) was 420. A couple of lines did not have the phenotypic records and their phenotypic values were replaced by the population mean for convenience of data analysis. A total of 38 microsatellite markers were used for the QTL mapping. These markers are more or less evenly distributed along five chromosomes with an average 10.8 centiMorgan (cM) per marker interval. The marker names and positions are given in the original article [23]. 

We inserted a pseudomarker in every 2 cM of the genome. Including the inserted pseudomarkers, the total number of loci subject to analysis was 200 (38 true markers plus 162 pseudomarkers). All the 200 putative loci were evaluated simultaneously in a single model. Therefore, the model for the short day flowering time trait is

(9) $$y=b_{0}+\overset{200}{\underset{k=1}{\sum }}X_{k}b_{k}+\varepsilon ,$$

where *X*_*k*_ is a 420 × 1 vector coded as 1 for one genotype and 0 for the other genotype for locus *k*. If locus *k* is a pseudomarker, *X*_*k*_ = Pr(genotype = 1), which is the conditional probabilities of marker *k* being of genotype 1. Finally, *b*_*k*_ is the QTL effect of locus *k*.

**Figure 8:**
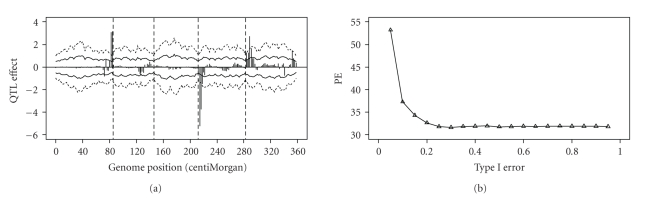
Result of the Arabidopsis data analysis. (a) Shows the estimated QTL effects for the entire genome and the empirical thresholds drawn from permutation within the Markov chain analysis at *α* = 0.05 (2.5%–97.5%, wider interval) and *α* = 0.10 (5%–95%, narrower interval). (b) Shows the plot of the squared prediction error (PE) against the Type I error obtained from the fivefold cross-validation test.

For the original data analysis, the burn-in period was 1000. The thinning rate was 10. The posterior sample size was 10000, and thus the total number of iterations was 1000 + 10000 × 10 = 101000. The posterior sample size of the within-chain permutation analysis was 80000, that is, 1000 + 80000 × 10 = 801000 iterations in total. The estimated QTL effects and the permutation generated 2.5%–97.5% and 5%–95% intervals are plotted in Figure 8(a). A total of 4 QTLs were detected on three chromosomes at *α* = 0.05. Chromosomes 1 and 4 each has one QTL and chromosome 5 has two QTL. When *α* = 0.10 was used, one more QTL on chromosome 1 was detected. 

The fivefold cross-validation shows that the optimal strategy of genome selection for this dataset was to include all QTLs in the model, regardless the significance of the estimated QTL effects (see Figure 8(b)). The general pattern of the PE profile remains the same as that of the simulated data. Below *γ* = 0.2 the decrease of PE was dramatic but after *γ* = 0.2 the PE values approached a stable value. 

### 3.8. Barley Data

The second data are the doubled haploid (DH) data obtained from Luo et al. [24]. This dataset consists of 150 doubled haploids (DHs) derived from the cross of two spring barley varieties, Steptoe and Morex, designated as the S × M cross. The phenotype was the spot blotch (a fungus *Cochliobolus sativus*) resistance measured as the lesion size on the leaves of barley seedlings. The total number of markers was 495 distributed along seven chromosomes of the barley genome. Because of the small sample size, we could not analyze all the 495 markers simultaneously (high collinearity). Therefore, we placed one pseudomarker in every 5 cM and overall obtained 225 pseudomarkers for the entire genome. The genotypes of the pseudomarkers were inferred from the multipoint method [25]. All the 225 putative loci were evaluated simultaneously in a single model. Therefore, the model for the disease resistance trait is

(10) $$y=b_{0}+\overset{225}{\underset{k=1}{\sum }}X_{k}b_{k}+\varepsilon ,$$

where *X*_*k*_ is a 150 × 1 vector coded as 1 for one genotype and 0 for the other genotype for locus *k*. If locus *k* is a pseudomarker, *X*_*k*_ = Pr(genotype = 1), which is the conditional probabilities of marker *k* being of genotype 1. Finally, *b*_*k*_ is the QTL effect of locus *k*.

**Figure 9:**
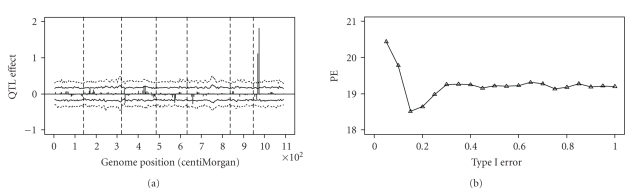
Result of the barley data analysis. (a) Shows the estimated QTL effects for the entire genome and the empirical thresholds drawn from permutation within the Markov chain analysis at *α* = 0.05 (2.5%–97.5%, wider interval) and *α* = 0.10 (5%–95%, narrower interval). (b) Shows the plot of the squared prediction error (PE) against the Type I error obtained from the cross-validation test.

The parameters of the MCMC experiment (e.g., burn-in period, thinning rate, etc.) were the same as the Arabidopsis data analysis. The estimated QTL effects and the permutation-generated 2.5%–97.5% and 5%–95% intervals are plotted in Figure 9(a). A total of two QTLs were detected on chromosome 7 at *α* = 0.05. These two are major QTL because their estimated values are way over the critical value. When the critical values at *a* = 0.10 were used, five more QTLs were declared as significant. 

The cross-validation analysis shows that the optimal strategy of genome selection for this dataset was to include all QTLs that are significant at *γ* = 0.15 (see Figure 9(b)). Below *γ* = 0.15 the decrease of PE was dramatic but after *γ* = 0.15 the PE values increased slightly until they reached a plateau at *γ* = 0.3 (see Figure 9(b)). This example demonstrated the usefulness of using cross-validation to select QTL for inclusion for prediction of genomic effect. 

### 3.9. Wheat Data

This example demonstrates the application of the Bayesian shrinkage analysis to QTL mapping for the number of seeded spikelets (a female fertility trait) in wheat. The experiment was conducted by Dou et al. [26] who made the data available to us for this analysis. A female sterile line XND126 and an elite cultivar Gaocheng 8901 with normal fertility were crossed for genetic analysis of female sterility measured as a quantitative trait. The *F*_1_ and *F*_2_ progeny of the parents were planted at the Huaian experimental station in China for the 2006-2007 growing season under the normal autumn sowing condition. The mapping population was an *F*_2_ family consisting of 243 individual plants. A total of 28 SSR markers were used in this experiment. These markers covered 5 chromosomes of the wheat genome with an average genome marker density of 15.5 cM per marker interval. The five chromosomes are only part of the wheat genome. These chromosomes were scanned for QTL of the fertility trait using the MCMC implemented Bayesian method. The dependent variable was the fertility phenotype while the independent variables were numerically coded genotype indicator variables for the part of genome under investigation. We placed one pseudomarker in every 5 centiMorgan (cM) of the genome. This generated 75 pseudomarkers for the five chromosomes. Therefore, we have a total of 75 independent variables. For each independent variable, the numerically coded value was the difference between the conditional probabilities of the two homozygote genotypes. Let *A*_1_ *A*_1_, *A*_1_ *A*_2_, and *A*_2_ *A*_2_ be the three genotypes for the *k*th pseudo marker of the genome. The numerically coded value for the locus is

(11) $$X_{jk}=p\left(G_{jk}=A_{1}A_{1}\;|\;\text{marker}\right)-p\left(G_{jk}=A_{2}A_{2}\;|\;\text{marker}\right)$$

for *k* = 1,…, 75. The map of the 75 pseudomarkers, the phenotypic values of the 243 plants, and the 75 numerically coded independent variables can be obtained from the authors.

**Figure 10:**
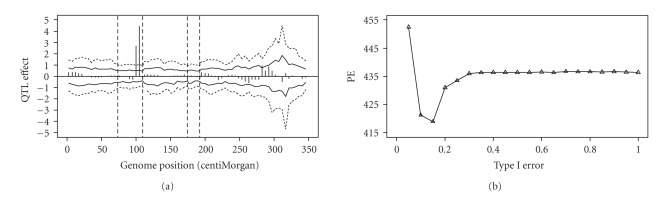
Result of the wheat data analysis. (a) Shows the estimated QTL effects for the entire genome and the empirical thresholds drawn from permutation within the Markov chain analysis at *α* = 0.05 (2.5%–97.5%, wider interval) and *α* = 0.10 (5%–95%, narrower interval). (b) Shows the plot of the squared prediction error (PE) against the Type I error obtained from the cross-validation test.

The parameters of the MCMC experiment (e.g., burn-in period, thinning rate, etc.) were the same as the previous two data analyses. The estimated QTL effects and the permutation-generated 2.5%–97.5% and 5%–95% intervals are plotted in Figure 10(a). A total of two QTL were detected on chromosome 2 at *α* = 0.05. When we lowered the critical value to *α* = 0.10, one more QTL was detected on chromosome 5. 

The cross-validation shows that the optimal strategy of genome selection for this dataset was to include all QTLs that are significant at *γ* = 0.1 (see Figure 10(b)). Below *γ* = 0.1, the decrease of PE was dramatic but after *γ* = 0.1 the PE values increased slightly until they reached a plateau at *γ* = 0.3.

In general, the optimal gamma value is somewhere between 0.1 to 0.2, but it varied from one experiment to another. The last two data analyses did indicate that including small QTL can be detrimental to genome selection. cross-validation is an experimental specific approach and is useful to decide how large a QTL should be included in the model for genome selection. 

## 4. Discussion

Bayesian shrinkage analysis can be used for both QTL mapping and genome selection. The two applications are quite different. QTL mapping aims to detecting QTL with large effects while genome selection tries to predict the total genetic values of individuals using markers of the entire genome. In QTL mapping, significance test is important, but Bayesian inference usually does not mix with significance test. This is because Bayesian inference focuses on the probability statement of a parameter given the information drawn from the current data and it does not intend to extend the statement beyond the data. Significance test, however, assumes a null distribution and tries to compare the statistics against the null distribution. The null distribution is purely hypothetical and, therefore, significance test gives conclusion that applies to hypothetical future experiments. The permutation test adopted in the Bayesian analysis is a convenient way to connect significance test with Bayesian analysis. Permutation analysis is a way to draw the null distribution. If a statistics, for example, estimated QTL effect, is far away from the null distribution, we are confident that this QTL is true. This type of significance test provides different conclusion from the Bayesian credible statement. In Bayesian analysis, people often report the *α*-equal-tail interval or *α*-highest posterior density (HPD) interval. These intervals cannot be used for significance test under the Bayesian shrinkage mapping. The reason is that almost all QTLs have an equal-tail interval covering the null value, for example, zero. Even the largest QTL in our simulation had a high probability mass at zero (see Figure 2). This zero-inflated posterior distribution for QTL effect is typical in Bayesian shrinkage mapping. If we had used the equal tail interval at *α* = 0.05 as the significance test criterion, only one QTL (the largest one), out of the 20 simulated QTLs, would have reached the statistical significance level. The permutation test, however, detected many major QTLs. 

In the simulation experiment, we observed that the percentile profiles for the 0.5 *α* × 100% − (1 − 0.5 *α*) × 100% interval were pretty much constant across the entire genome (see Figures 3 and 4). This is due to the uniform information content across the genome. We simulated 241 markers covering the entire genome evenly with 10 cM per marker interval. These markers were codominant with no missing genotypes. In contrast, the three real data analyses showed that the percentile lines varied dramatically across the genome. The intervals were narrow at marker positions and wide when the positions are away from the markers. The lengths of marker intervals also varied across the genome, making the information content much uneven across the genome. The location specific empirical threshold values in real data analysis mean that different locations of the genome should use different criteria for QTL detection. We have two QTL with the same estimated effect but located in different regions of the genome, one may be declared as significant but the other may not be significant due to the variation in information content. This actually justifies the use of estimated QTL effects, not some kind of test statistics, for significance test. 

In classical QTL mapping experiments, investigators always use some kinds of test statistics (e.g., *t*-test, *F*-test, likelihood ratio test, or LOD score) to decide whether a QTL is significant or not. A permutation test also draws critical values for the test statistic under consideration, not the critical values for the QTL effects. This merely reflects the tradition or convention of people who do statistical analysis and does not mean that a test statistic is the only quantity that can be used in QTL mapping. The reason for using test statistics is that one can compare the observed test statistic (calculated values) with the critical values of some distribution, for example, normal distribution, *F*-distribution, *t*-distribution, and chi-square distribution. The critical values of these standard distributions can be found from statistical tables or calculated from statistical analysis software. With the permutation test, we never need the critical values of the standard distributions. Therefore, there is no need to use the test statistics. Directly comparing the estimated QTL effects with the critical values is more intuitive. 

Significance test can help us decide which QTL should be claimed as significance. The significant QTL will be the targets for further study, for example, cloning or marker assisted selection. What do we do with those QTLs whose effects do not reach the significance level? These QTLs may not be significant individually, but collectively they may contribute to a large proportion of the phenotypic variance. This implies that they are perhaps useful to predict the total genetic effects of individuals [18], a technology called genome selection. Our cross-validation experiments showed that QTLs should be used to predict the total genetic effects once they reached a certain critical value. Including many small QTLs can be harmful to genome selection. Common sense tells us that estimated effects of small QTLs are most likely caused by noises rather than by true signals and inclusion of the many small QTLs to predict the genetic effects may be even worse than inclusion of only the significant QTL.

## 5. Conclusions

We developed two permutation tests, one is called “permutation outside Markov chain” and the other is called “permutation inside Markov chain”. The latter is recommended because it is (1) faster computationally and (2) slightly more conservative. The empirical thresholds can be used to detect QTL. cross-validation studies showed that small QTLs should be excluded from the model for prediction of the total genetic effects for individual plants. The criterion of exclusion is experimental specific and should be decided by cross-validation.

## Figures and Tables

**Table 1:** QTL parameters used in the simulation experiment.

QTL	Position	Marker	Effect	Prop-G	Prop-P
1	50	11	4.47	0.1505	0.1308
2	125	26	3.16	0.0752	0.0654
3	205	42	−2.24	0.0378	0.0328
4	235	48	−1.58	0.0188	0.0163
5	355	72	2.24	0.0378	0.0328
6	360	73	3.16	0.0752	0.0654
7	610	123	1.10	0.0091	0.0079
8	630	127	−1.10	0.0091	0.0079
9	800	161	0.77	0.0045	0.0039
10	900	181	1.73	0.0225	0.0196
11	905	182	3.81	0.1093	0.0950
12	920	185	2.25	0.0381	0.0331
13	1100	221	−1.30	0.0127	0.0111
14	1210	243	−1.00	0.0075	0.0065
15	1305	262	−2.24	0.0378	0.0328
16	1335	268	1.58	0.0188	0.0163
17	1345	270	1.00	0.0075	0.0065
18	1365	274	−1.73	0.0225	0.0196
19	1800	361	0.71	0.0038	0.0033
20	2300	461	0.89	0.0060	0.0052

Prop-G means the proportion of genetic variance contributed by the QTL and Prop-P means the proportion of phenotypic variance contributed by the QTL.
